# Clinical Presentation of Peyronie’s Disease: A Retrospective Study of 564 Cases

**DOI:** 10.3390/diagnostics14111125

**Published:** 2024-05-29

**Authors:** Gianni Paulis, Giovanni De Giorgio, Andrea Paulis

**Affiliations:** 1 Department of Urology and Andrology, Peyronie’s Care Center, Castelfidardo Clinical Analysis Center, 00185 Rome, Italy; 2 Section of Ultrasound Diagnostics, Department of Urology and Andrology, Castelfidardo Clinical Analysis Center, 00185 Rome, Italy; g.degiorgio@analisiclinichecastelfidardo.it; 3 Bambino Gesu’ Children’s Research Hospital, IRCCS (Istituti di Ricovero e Cura a Carattere Scientifico), 00165 Rome, Italy; andrea.fx.94@gmail.com; 4 Neurosystem for Applied Psychology and Neuroscience, Janet Clinical Centre, 00195 Rome, Italy

**Keywords:** anxiety, depression, erectile dysfunction, pain assessment, Peyronie’s disease

## Abstract

Peyronie’s disease (PD) affects the penile albuginea, resulting in penile deformity, pain, erectile dysfunction (ED), and an anxious–depressive state. PD diagnosis involves a thorough medical history, penile palpation, documentation of the penile deformation, a dynamic penile echo color Doppler ultrasound (PCDU), and the completion of questionnaires for the evaluation of pain, ED, and psychometric tests. The aim of this study was to evaluate the symptoms of PD and their prevalence in PD patients in the active phase who had access to our andrology clinic. Inclusion criteria: availability of data on patients diagnosed with PD, including detailed medical history, blood tests, penile palpation, photographic documentation of penile deformity, and penile PCDU. Exclusion criteria: PD patients in the stable phase or those without the specified tests and data mentioned above. Our study found a higher prevalence of PD in younger patients (24.2%), a higher coexistence of PD with chronic prostatitis (35.6%), a higher percentage of cases of association between penile deformity and penile curvature (84.4%), a higher prevalence of “significant anxiety” (88.4%), a higher presence of plaque calcification (35.6%), and the detection of a longer duration of the first phase of PD (>18 months). The most frequently observed type of penile curvature was dorsal, followed by left lateral, right lateral, and, less commonly, ventral. We observed a significant statistical correlation between patient age and IIEF score, indicating that patients over the age of 40 years are at a higher risk of experiencing ED. We found a strong statistical relationship between VAS score and age. As age increases, the VAS score decreases, suggesting that younger patients reported more penile pain compared to those who were older than 40 years. Furthermore, we found that penile pain has a significant impact on the psychological state of PD patients. We also found that 38.8% of PD patients suffered from severe anxiety. In relation to this, psychotherapy should be integrated into PD treatment to improve the quality of life and treatment adherence.

## 1. Introduction

Peyronie’s disease (PD) is a pathological condition characterized by inflammation affecting the tunica albuginea of the penis, which leads to the formation of a non-elastic fibrous tissue that deforms the penis. PD only affects genetically predisposed males [1,2]. PD affects males with a prevalence ranging from 3.2 to 13% in the Western world, while in East Asian countries it is less common, with levels of prevalence ranging from 0.6 to 5% [3,4,5,6,7,8]. In black populations originating from Africa, the prevalence of PD appears to be even lower, with percentages ranging from 0.1 to 3.5% [9,10,11].

Although the pathogenesis is not yet completely known, the traumatic theory is generally accepted within the scientific literature. When the penis is subjected to a trauma or repeated microtraumas, this/these will cause a small hematoma at the level of the tunica albuginea, which, instead of being reabsorbed, in the presence of an abnormal wound healing disorder of the connective tissue, will cause an excessive deposition of collagen, with the consequent formation of a fibrous plaque that is inelastic and capable of deforming the penis [12,13,14]. In the scientific literature, several articles have appeared in the 21st century that have clarified the important role that “oxidative stress” plays in the pathophysiology of PD [15,16,17,18,19,20,21,22]. There are essentially four symptoms of PD: penile deformity, either as curvature or as simple deformation, associated or not with curvature (shortening, indentation, hourglass shape, hinge effect, flail penis, penile torsion); penile pain; erectile dysfunction (ED); and an anxious–depressive state [23,24,25,26,27,28]. Regarding the symptoms, some authors have rightly described this disease as “a psychologically and physically devastating disorder …” as all these symptoms can indeed have a great impact on the physical and mental health of affected patients [29]. 

PD progresses in two main stages. The first phase typically lasts between 12 and 18 months; this phase is the “active inflammatory phase”, in which plaque forms and progresses to fibrosis and potential calcification, accompanied by pain and worsening of the penile deformity. In the second phase, known as the chronic “stabilized phase”, penile pain decreases and the plaque size and deformity stabilize [2,15,19,21,22,30,31]. 

The second phase of PD usually starts 12 to 18 months after the disease first appears [31]. The therapeutic option indicated for the first phase of PD is conservative medical treatment, which includes oral medications, iontophoresis, intralesional injections, topical creams, shock wave therapy, vacuum penile therapy, and a traction device [32,33,34,35,36,37,38,39,40,41,42,43,44,45]. Surgery is recommended during the second phase of the disease, when the curvature is significant and/or when there is severe ED [46,47,48,49,50,51]. 

The diagnosis of Peyronie’s disease includes penile palpation, photographic documentation of the penile deformation (following Kelâmi’s guidelines), dynamic penile echo color Doppler ultrasound (PCDU), and the completion of questionnaires such as a visual analog scale (VAS) for pain assessment, the International Index of Erectile Function (IIEF) for evaluating erectile function, and psychometric tests like the Peyronie’s Disease Questionnaire (PDQ) [52,53,54,55,56,57,58,59,60,61]. Having a large case series of Parkinson’s disease patients that we followed in an outpatient setting, we conducted this study in order to obtain more reliable results due to its significant statistical power. The aim of this study was to evaluate the main symptoms of PD and their prevalence in PD patients in the active phase who had access to our andrology clinic.

## 2. Materials and Methods

### 2.1. Study Population

We conducted a retrospective analysis of the clinical database of our andrology clinic. We identified 564 patients with PD who had been seen at our Peyronie’s care center from January 2013 to February 2024. This study was carried out in accordance with the principles outlined in the Declaration of Helsinki (2013). All participants were contacted and gave their informed consent for this study. However, sensitive data were anonymized in accordance with privacy regulations as per Legislative Decree 10 August 2018, n. 101, adapted to the GDPR.

### 2.2. Inclusion Criteria

(1). Patients with PD in the active phase. 

(2). Availability of the following data on patients found to be affected by PD: in-depth medical history (including all diseases, cigarette smoking, consumption of alcohol, history of recent penile trauma, previous endourological maneuvers, previous radical retropubic prostatectomy); blood tests: basal blood sugar, glycosylated hemoglobin, cholesterol, triglycerides, and homocysteinemia; penile palpation; photographic documentation of penile deformation (following Kelâmi’s guidelines); dynamic penile echo color Doppler ultrasound (PCDU); and the completion of questionnaires such as a visual analog scale (VAS) for pain assessment, the International Index of Erectile Function (IIEF) for evaluating erectile function, and psychometric questionnaires such as the Generalized Anxiety Disorder-7 questionnaire (GAD-7, for the assessment of anxiety), the Patient Health Questionnaire-9 (PHQ-9, for the assessment of depression), and the Peyronie’s Disease Questionnaire (PDQ) symptom bother, for assessing the psychosexual impact of PD.

### 2.3. Exclusion Criteria 

(1). Patients with PD in the stabilized phase (stable penile curvature for at least 6 months, stable penile plaque volume for at least 6 months). 

(2). All patients without the specified test data indicated above. 

### 2.4. Clinical Data

The medical records related to the first visit of patients suspected of having PD to our clinic were analyzed. Clinical data (including the presence of concomitant diseases and cigarette smoking, consumption of alcohol, history of recent penile trauma, previous endourological maneuvers, and previous radical retropubic prostatectomy) were collected from the medical records of the 564 PD patients. Clinical data of these patients were reviewed to identify the results of basal blood sugar, glycosylated hemoglobin (hemoglobin A1c), cholesterol, triglycerides, and homocysteinemia. Additionally, this study analyzed the outcomes of penile palpation, photographic documentation of penile deformation (including the goniometric measurement of angulation following Kelâmi’s guidelines), an evaluation of potential multiplanarity of curvature, and dynamic PCDU for the detection of plaque and potential ED, the measurement of its dimensions, and the calculation of its volume (mm^3^) using an ellipsoid formula (volume = 0.524 × width × length × thickness) [52,53,54,55]. Furthermore, this exam served to identify calcifications and their sizes. 

All patients completed the following questionnaires: a visual analog scale (VAS) questionnaire for pain assessment; the International Index of Erectile Function (IIEF) for the evaluation of erectile function; and three validated psychometric tests: the Generalized Anxiety Disorder-7 questionnaire (GAD-7, for the assessment of anxiety), the Patient Health Questionnaire-9 (PHQ-9, for the assessment of depression), and the Peyronie’s Disease Questionnaire (PDQ) symptom bother, to evaluate the psychosexual impact caused by PD.

The VAS questionnaire consists of a 10 cm line on paper, where each 1 cm point represents a level of pain intensity; patients mark the level of pain perceived on this line. Scores range from 0 (no pain) to 10 (most severe pain). We classified the VAS scores as follows: 1–5 for mild to moderate pain, 6–7 for severe pain, and 8–10 for very severe pain [56]. The IIEF Erectile Function Questionnaire, used to evaluate erectile dysfunction, includes 15 questions with 5 response options for a total score ranging from 0 to 30. The interpretation of the score is as follows: severe ED (0–10), moderate ED (11–16), mild to moderate ED (17–21), mild ED (22–25), and no erectile dysfunction (26–30) [56]. ED was considered present if the score was <26 [57]. The GAD-7 anxiety questionnaire consists of seven questions with four response options, with a score ranging from 0 to 21. Anxiety levels were classified as follows: minimal anxiety (0–4), mild anxiety (5–9), moderate anxiety (10–14), and severe anxiety (15–21). “Significant” anxiety was defined as a GAD-7 score >9 [58]. The PHQ-9 questionnaire, composed of 9 questions with 4 response options, produces a score range between 0 and 27. The severity of depression was classified as minimal (0–4), mild (5–9), moderate (10–14), moderately severe (15–19), or severe (20–27) [59]. “Significant” depression was considered present if the PHQ-9 score was >9 (indicating moderate to severe depression) [59]. The PDQ assesses the severity and impact of symptoms related to Peyronie’s disease in three areas: psychological and physical symptoms, penile pain, and symptom bother. We only used the third part (PDQ—symptom bother) because we had already used other tests (IIEF, VAS, GAD-7, and PHQ-9) for the other PD symptoms. Answers were rated on a scale from 0 to 4, resulting in a total score ranging from 0 to 16 [60,61].

The size of the plaque calcification, measured in millimeters, was classified into three groups based on the maximum size of the calcified area: grade 1 (punctate or ≤3 mm), grade 2 (>3 mm and <15 mm), and grade 3 (≥15 mm or ≥2 plaque >10 mm), following the classification of Levine et al. [62]. 

After identifying the penile curvature and measuring the relative degree of curve, the curvatures were divided into 4 categories. This is our modification of the Kelâmi classification; we divided the first class of the Kelâmi classification into two sub-categories. First category: curvature < 15 degrees; second category < 30 degrees; third category: curvature between 30 and 60 degrees; fourth category: curvature > 60 degrees. The original classification of penile curvatures by Kelâmi is as follows: first class, curvature < 30 degrees; second class, curvature between 30 and 60 degrees; third class, curvature > 60 degrees [52,53].

### 2.5. Study Endpoints 

The primary endpoints of this study were as follows:

- Identification of demographics, clinical characteristics, and lifestyle habits related to excessive alcohol and smoking consumption in patients with PD;
- Research on the frequency of younger patients (under 40 years of age);
- Research into the time (expressed in months) between the onset of PD and the diagnosis of the disease;
- The identification of the main symptoms of PD and the assessment of their severity by means of photography of the penis and goniometric measurement of the angle; VAS questionnaire for pain; dynamic penile eco-color Doppler ultrasound and IIEF questionnaire for the evaluation of erectile function; GAD-7 and PHQ-9 for the assessment of anxiety and depression, respectively; and PDQ bother score to assess the psychosexual impact of PD;
- Study of penile plaque with regard to ultrasound appearance, palpation characteristics, volume, location, and possible multifocality;
- Study of the types of echogenicity of plaque in relation to their palpability and plaque volume;
- Study of the types of echogenicity of plaque relative to their palpability and onset of the disease.
- The secondary endpoints in this study were as follows:
- Search for a possible correlation between VAS score and patient age;
- Search for a possible correlation between the presence (and degree) of penile curvature and patient age;
- Research on the possible correlation between plaque volume of PD and the time elapsed since the disease onset;
- Research on the possible correlation between the degree of penile curvature and time elapsed since the disease onset;
- Research on the possible correlation between IIEF score and time elapsed since the disease onset;
- Research on the possible correlation between VAS score and time elapsed since the disease onset;
- Research on the possible correlation between the degree of penile curvature and PD plaque volume;
- Research on the possible correlation between IIEF score and PD plaque volume;
- Research on the possible correlation between IIEF score and patient age;
- Research on the possible correlation between GAD-7 score and PHQ-9 score;
- Research on the possible correlation between GAD-7 score and PDQ bother score;
- Research on the possible correlation between PHQ-9 score and PDQ bother score;
- Study of the potential relationship between the intensity of anxiety (GAD-7 score) and severity of penile curvature;
- Study of the potential relationship between the intensity of depression (PHQ-9) and severity of penile curvature;
- Study of the potential relationship between the intensity of PDQ bother (psychosexual impact of PD) and severity of penile curvature;
- Study of the potential relationship between the VAS score and GAD-7 score;
- Study of the potential relationship between the VAS score and PHQ-9 score;
- Study of the potential relationship between the VAS score and PDQ bother score;
- Research on the possible correlation between the IIEF score and GAD-7 score;
- Research on the possible correlation between the IIEF score and PHQ-9 score;
- Research on the possible correlation between the IIEF score and PDQ bother score (psychosexual impact of PD);
- Research on the possible correlation between the IIEF score and severity of penile curvature;
- Research on the possible correlation between the severity of ED (IIEF score) and VAS score (severity of penile pain).

### 2.6. Statistical Analysis

We used CalculatorSoup^®^ software (version of 7 March 2023, Ashland, MA, USA) to perform statistical analysis, which included calculating the standard deviation and mean. We utilized MedCalc statistical software (MedCalc Software Ltd., Version 22.023, 2023, Ostend, Belgium) to conduct a two-tailed chi-squared test and unpaired *t*-test. The Pearson correlation coefficient was computed using Statistics Kingdom statistical software (version 2017, Melbourne, VIC, Australia, http://www.statskingdom.com accessed on 28 May 2024) and Excel (version 2011, MS Office, Redmond, WA, USA). We conducted a post hoc analysis to evaluate the statistical power achieved using G*Power 3.1 software (version for Mac and Windows, 6 February 2019-Release 3.1.9.4, Düsseldorf, Germany). A significance level of 5% for alpha error (*p*-value < 0.05) was used in the statistical analyses to determine statistical significance.

## 3. Results

The 564 patients with Peyronie’s disease were aged between 21 and 74 years, with a mean age of 49.54 years (SD ± 12.25). The 137 younger patients, with an age up to 40 years (mean age 32 years ± 5.04), represented 24.2% of the total patients. The 427 patients aged over 40 years (mean age 60.52 years, SD ± 7.77) represented 75.7% of the PD patients. All 564 patients, during their first visit to our clinic, reported that in the last 6 months their symptoms had been worsening, particularly penile curvature and penile pain (when present).

Table 1 and Table 2 present the demographic and clinical characteristics of the PD patients. The four main symptoms of PD (and their prevalence) and the indication of the time elapsed since the onset of the disease are shown in Table 2. 

We found ED in 225 cases (39.8% of all PD patients). Of these, 127 patients (56.4%) reported experiencing ED before developing PD. The results related to the timing of ED presentation in PD patients are shown in Table 3.

Table 4 displays the results concerning the ultrasound appearances, palpation characteristics, and volumes and locations of penile plaque.

We detected multifocal penile plaque in 91 out of 564 cases (16.1% of cases).

Table 5 shows the frequencies of various severity categories of curvature and the results regarding the frequencies and types of penile deformities in the 564 patients with PD.

Herein, we present our results regarding the echogenicity of the plaque relative to palpability and plaque volume. Penile plaque with a volume of less than 300 mm^3^ (ranging from 66.7 to 295,749 mm^3^, mean volume 192.7 mm^3^, SD ± 66.4) was found in 66 cases. The plaque was non-palpable in 53 cases with the following echogenicity: 17 cases hypo-isoechoic, 32 cases hyperechoic, and 4 cases mixed associated with calcification. In the remaining 13 cases, the plaque was palpable with the following echogenicity: 2 cases hypo-isoechoic and 11 cases isoechoic. Penile plaque with a volume greater than 300 mm^3^ (ranging from 302.11 to 3657.7, mean volume 872.3 mm^3^, SD ± 615.8) was found in 498 cases. Of this plaque, nine cases were non-palpable with the following echogenicity: one case hypo-isoechoic, one case isoechoic, one case iso-hyperechoic, and six cases hyperechoic. In the remaining 489 cases, the plaque was palpable with the following echogenicity: 7 cases hypoechoic, 11 cases isoechoic, 268 cases hyperechoic, 5 cases hypo-isoechoic-hyperechoic, 1 case iso-hyperechoic, and 197 cases mixed associated with calcification. Findings related to the ultrasound appearance, palpation characteristics, and plaque volume are shown in Table 4. 

Here, we present our results regarding the echogenicity of the plaque relative to its palpability and the onset of the disease. Of the 409 cases with disease onset from 6 to 12 months, the plaque was not palpable in 62 cases and the echogenicity was as follows: hypoechoic in 20 cases, isoechoic in 12 cases, iso-hyperechoic in 1 case, and hyperechoic in 29 cases. In the other 347 cases, the plaque was palpable and the echogenicity was as follows: isoechoic in 11 cases, hyperechoic in 210 cases, hypo-isoechoic in 7 cases, hypo-isoechoic-hyperechoic in 3 cases, iso-hyperechoic in 1 case, and mixed in 115 cases with associated calcification. In 155 cases with onset of the disease from 13 to 26 months, the related plaque was always palpable and the echogenicity was as follows: hypo-isoechoic–hyperechoic in 2 cases, hyperechoic in 67 cases, and mixed associated with calcification in 86 cases. None of the plaque was completely calcified on the ultrasound, and none of the plaque was in the stabilizing phase. Our findings related to the disease onset are shown in Table 2. Findings related to the ultrasound appearance and palpation characteristics are shown in Table 4.

Regarding the psychological states of the 564 PD patients, the psychosexual impact (PDQ bother) of PD has already been shown in Table 2, and the prevalence of an anxious–depressive state is shown in Table 6.

We found a statistically significant correlation (Pearson coefficient) between the VAS score and age. As age increased, the VAS score decreased, so in younger patients, penile pain was higher compared to patients over 40 years old. Furthermore, our comparison of the respective means of the VAS scores (cases with age *≤* 40 years vs. cases with age > 40 years) revealed a statistically significant result; with an unpaired t-test, the 95% confidence interval of this difference was from 0.167527566 to 1.046758034, with a two-tailed *p* value = 0.0070 (<0.05).

**Figure 1:**
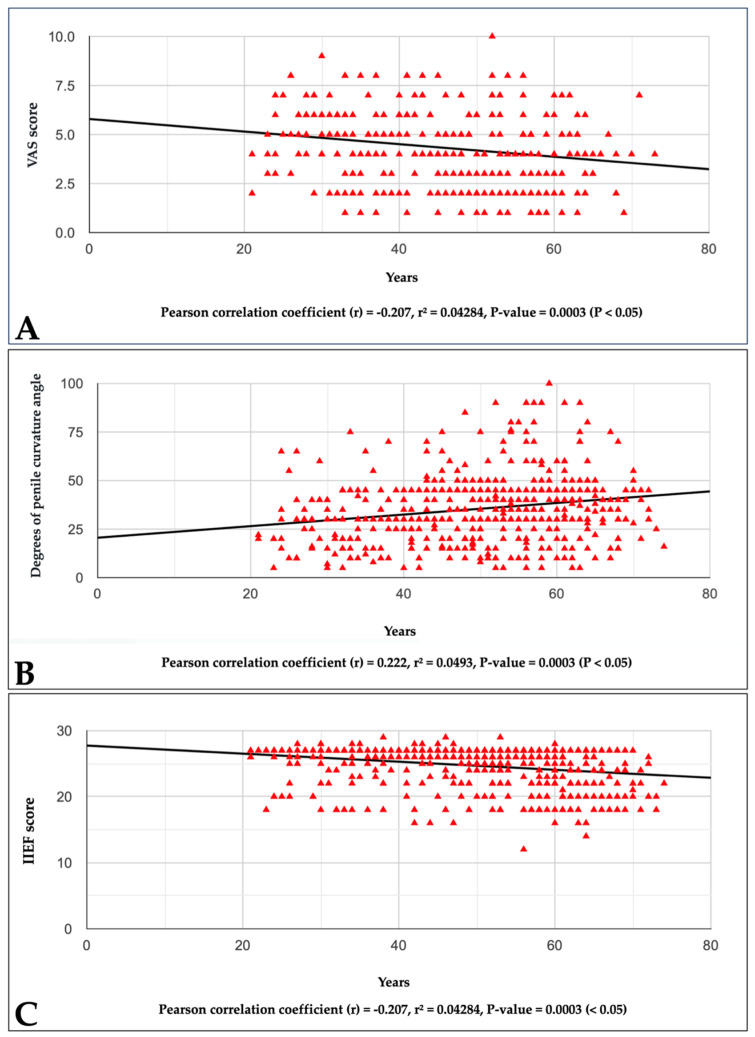
Graphs showing the respective correlations between VAS score (**A**), degree of penile curvature angle (**B**), IIEF score (**C**), and the age of a patient with Peyronie’s disease.

The statistical correlation is illustrated in Figure 1A, and it is shown in Table 7.

We found a statistically significant correlation (Pearson coefficient) between the degree of penile curvature and patient age. This showed that the degree of curvature was greater in patients over 40 years old. Furthermore, as evidence of this, our comparison of the respective means of the degrees of penile curvature angles (cases with age *≤* 40 years vs. cases with age > 40 years) revealed a statistically significant result: unpaired *t*-test: 95% confidence interval of this difference from 34.18480287 to 41.32120713, two-tailed *p* value = 0.0001 (<0.05). The statistical correlation is illustrated in Figure 1B, and it is shown in Table 7.

We found a significant statistical correlation (Pearson coefficient) between the IIEF score and patient age. With a greater patient age, the IIEF score tended to decrease, indicating that patients aged over 40 years had a greater degree of severity of erectile dysfunction than those aged 40 years or younger. The statistical correlation is illustrated in Figure 1C, and it is shown in Table 7.

We did not find a statistically significant correlation (Pearson coefficient) between IIEF score and PD plaque volume. The statistical correlation is shown in Table 7, among all possible statistical correlations between the different variables.

The current study was found to have a statistical power of 100% to detect an effect size (w) of 0.20 with two degrees of freedom (α = 0.05).

## 4. Discussion

In our results, the mean age of the PD patients was 49.54 years, which is similar to some ages previously reported, averaging 48.2–49.6 years [5,63,64]. Other authors, however, have reported an average age of PD patients of between 52 and 57 years [3,53,65,66]. We believe that this difference is due to the fact that the latter studies were published many years earlier, and the average age of PD patients has decreased, probably due to the increasing incidence of PD in younger patients. Indeed, certain authors have observed an increase in the incidence of PD among young patients, with a recent report of 18.6% of subjects aged 40 years or younger compared to 16.9% of PD patients aged 40 years or younger in a study conducted approximately 9 years ago [64,67]. In the current study, which included a larger sample, we detected a still higher incidence of PD in younger patients, at 24.2% of the total patients aged 40 years or younger (see Table 1).

We also found a higher prevalence of certain clinical conditions associated with PD in our study compared to those reported in the general population in the scientific literature, including chronic prostatitis (35.6% versus 8.2%), hypothyroidism (4.7% versus 0.6–1.09%), and Ledderhose disease (2.6% versus 0.001–0.00175%) [68,69,70,71]. In our results, we found penile curvature in 90.6% of cases, which is similar to the findings of other studies on PD patients reported in the literature, which detected penile curvature in 87.6–94% of cases [25,27,72].

The most frequent type of penile deformity detected was penile shortening (498 cases, 88.2%). In 119 cases (23.4%), two types of penile deformity were found in the same patient. In 389 cases (68.9%), the penile deformity was found to not be associated with any other type of penile deformity. When there was an association, penile deformity was often associated with penile curvature (49 cases, 84.4%). These results are quite different from those obtained by Kadioglu, who observed penile deformation associated with curvature in 52.4% of cases and solitary penile deformation in 12.3% of cases [27]. Other conflicting data when compared with ours are those of Cakan et al. who, in their study on PD patients, observed a prevalence of 13% of a “pure deformity” without penile curvature [73]. Regarding the type of penile curvature, in our study, dorsal curvature was the most commonly observed type, followed by left lateral curvature, right lateral curvature, and, less commonly, ventral curvature. Similar results have been described in numerous studies and reviews already published [25,53,63,72,73,74,75,76]. Regarding the angle of penile curvature, we agree with other authors that the measurement of the angle should be carried out directly by the uro-andrologist because patients suffering from Peyronie’s disease tend to overestimate their own degree of curvature of the penis [53].

We observed the presence of penile pain in 53.3% of cases in our study. In the scientific literature, the prevalence of penile pain has been observed by various authors, with percentages ranging from 17 to 70% [25,53,72,73,74,75]. Additionally, we discovered a highly significant statistical correlation between the VAS score and age. As age rose, the VAS score declined, indicating that younger patients experienced higher levels of penile pain compared to those over 40 years old.

In our case series, we found erectile dysfunction (ED) in 39.8% of cases. In the scientific literature, the prevalence of ED has been observed by various authors, with percentages ranging from 15 to 60.1% [23,27,72,73,74,75]. We observed a significant statistical correlation between the patient age and IIEF score. The IIEF score was typically lower in older patients (aged > 40 years), indicating that these patients are at a higher risk of experiencing ED compared to those aged ≤ 40 years.

Previously, researchers have observed that, in some cases, ED can occur before the onset of PD, with a prevalence of between 39.8% and 57.6% [77,78,79]. Accordingly, in our study, 56.4% of patients (127 out of the 225 total patients with PD + ED) reported experiencing ED before developing PD.

In our research, the prevalence of “significant anxiety” (88.4%) was higher than the rates reported in other studies in the literature that refer to “distress” and “emotional” concerns (80–81% of cases) [80,81]. For instance, Levine’s study found that 80.1% of PD patients experienced “emotional distress” [62].

The prevalence of “significant depression” found in our study (57.6%) was higher than that documented in Nelson’s studies (48%) [26,82]. The higher number of PD cases in our study (564 cases) compared to that in Nelson’s study (92 cases) likely explains the disparity in results. In our research, we also found a slightly higher psychosexual impact (PDQ bother mean score = 8.8) compared to those reported by other authors (6.3–8.0) [60,64,83].

The scores obtained from the three psychometric questionnaires (GAD-7, PHQ-9, and PDQ-bother) completed by all PD patients revealed a significant impact of the disease on their psychological state. Moreover, we found a statistically significant correlation between the VAS score and all the scores obtained with the three psychometric tests mentioned above. In the literature on this topic, we found consistent results [60,84].

In addition, we found a statistically significant correlation between the severity of ED (IIEF score) and the scores for PD patients on the three psychometric tests. Some articles in the literature on this topic support this finding [60,84].

Furthermore, we found a statistically significant correlation between the severity of penile curvature and all the scores for PD patients on the three psychometric tests. Consistent results were found in the literature on this topic [60,64,84].

In our series, plaque was not palpable in 10.9% of cases. This fits with previous reports from the scientific literature, where in some studies the percentages of non-palpable plaque ranged from 2.8 to 22.0% of cases [53,76,85,86]. We detected the presence of calcification in 35.6% of cases. This was slightly higher than expected, as other studies in the literature have found that approximately 20–31.4% of men with PD have plaque calcification [87,88,89]. According to Levine et al.’s classification, in our study, the plaque was Grade 1 in 11.4% of cases, Grade 2 in 64.6%, and Grade 3 in 23.8% of cases [62]. Grade 1 was less represented than we had anticipated, as Levine et al., in a case series of 1041 PD patients, found plaque calcifications in 27.2% of cases, with the categories represented as follows: Grade 1 in 40.8% of cases, Grade 2 in 27.6%, and Grade 3 in 31.6% of cases [62].

Regarding the location of the penile plaque, in our case series, we found the following penile locations with their respective levels of prevalence: proximal third 12.4%, middle third 21.9%, distal third 30.3%, and mixed location 35.2%. This placed fewer in the proximal third and more in a mixed location than we had expected, since Byström et al. previously found the following penile locations with different levels of prevalence: proximal third 41.0%, middle third 26.0%, distal third 26%, mixed location 7.0% [90]. However, we propose that these data were very different because they were collected in the year 1976 and solely through palpation, without the use of penile ultrasound. Today, thanks to the technical evolution of ultrasound machines and with the help of elastography, it is possible to identify non-palpable penile plaque [91,92,93,94,95,96,97].

We also found a statistically significant relationship between the degree of penile curvature and patient age, with the degree of curvature greater in patients over 40 years. We did not find similar information on this topic in the literature.

In another finding, we discovered a significant statistical correlation between the degree of penile curvature and the penile plaque volume. Other authors have not found such a correlation [98]. However, we believe that their different conclusions arose since those authors did not assess the volume of the plaque in three dimensions using an ellipsoid formula; instead, they only determined the plaque size by evaluating the length of the plaque in centimeters.

We also found a statistically significant correlation between the degree of penile curvature and the time since the disease onset. Additionally, we found a highly significant statistical correlation between the penile plaque volume and the time since the disease onset. We believe that these results reflect the natural history of Peyronie’s disease. However, we found no statistically significant correlation between the IIEF score and the time since the disease onset. Similarly, we found no statistically significant correlation between the VAS score and the time since the disease onset. We believe that the lack of correlation in these cases may be due to the fact that ED and penile pain are not always present during Peyronie’s disease. We also did not observe a statistically significant correlation between the severity of ED (as measured by the IIEF score) and the severity of penile curvature. In the literature, we found similar results in a study by Sereflogu et al. [84]. Furthermore, we did not find a statistically significant correlation between the severity of ED (IIEF score) and the severity of penile pain (VAS score), marking a difference from the results reported in the study by Sereflogu et al. [84].

Lastly, in our study, in which all patients were in the first phase of PD, we found that the disease onset occurred between 6 and 26 months before our PD diagnosis. In the scientific literature, it is reported that PD generally stabilizes at between 12 and 18 months from the onset of the disease [15,31,34,51,99,100]. However, in our results, we noted that the first phase (active phase) of PD could last beyond 18 months. In fact, all patients with PD, even those with a disease onset beyond 18 months, still showed signs of active disease, with penile curvature continuing to worsen and penile pain, when present from the beginning, continuing to persist. Additionally, we found that, in the five patients with PD who had an onset of the disease at beyond 18 months (from 22 to 26 months), penile pain was already absent at the time of our diagnosis. Nevertheless, their penile curvature continued to increase, indicating that PD was still in the active phase. In all five cases, the plaque was hyperechoic on our ultrasound and did not show any calcification.

In all the studies mentioned in our article where the statistical power (post hoc analysis evaluation) of the study was calculated, it was found to be 99% or 100%.

In summary, this study adds to the existing literature on PD by finding a higher prevalence of the disease in younger patients (24.2%), a higher coexistence of chronic prostatitis (35.6%), a higher percentage of association of penile deformity with penile curvature (84.4%), a higher prevalence of “significant anxiety” (88.4%), a greater presence of plaque calcification (35.6%), and the detection of a longer duration of the first phase of the disease of more than 18 months. It is possible that both our study and the other studies mentioned may be susceptible to some bias. We believe this is possible because there is still a gap in knowledge, for example, regarding the duration of the initial phase of the disease, etc. Many studies cited in our research were conducted many years ago, and the symptom evaluations were performed using the diagnostic tools available at that time, even though they had a good statistical sample. Additionally, in most cases, the size of the plaque was only assessed by measuring the length of the plaque and not by calculating the volume of the plaque. For Peyronie’s disease, there is undoubtedly a gap in knowledge that mainly concerns its etiopathogenesis, partly hypothesized (such as the traumatic hypothesis) and partly known (involving the overproduction of cytokines and free radicals). The genetic cause and reasons for differences in prevalence among various human demographic groups have not been clarified, nor the exact duration of the two phases of the disease. There is also a lack of complete agreement on the most appropriate conservative medical therapy to use in the first stage of the disease. The uro-andrological scientific community needs to work on filling these knowledge gaps in order to offer the best and most reliable treatment to patients with PD, leading to the best clinical response.

## 5. Conclusions

An accurate medical history and diagnostic tests, including penile curvature identification, dynamic penile ultrasound, VAS, IIEF, and psychological assessments, are essential for the appropriate management of Peyronie’s disease. Here, we observed a greater prevalence than expected of PD in younger patients (24.2% of cases). The most frequently observed type of penile curvature was dorsal, followed by left lateral, right lateral, and, less commonly, ventral. Penile shortening was the most common deformity found, followed by the penile hourglass, divot, torsion, and flail penis. In almost a quarter of cases, patients had more than one type of deformity. Penile curvature was frequently linked with these deformities. PD patients over the age of 40 years were at a higher risk of experiencing ED compared to those younger than or at 40 years. Of our PD patients with ED, 56.4% reported experiencing this problem before developing PD. We noted that the first phase of PD could last beyond 18 months. Younger PD patients reported more penile pain compared to those who were older than 40 years, and penile pain had a strong impact on the psychological state of patients. High rates of significant anxiety and depression were present in patients with PD, and 38.8% of PD patients suffered from “severe” anxiety. Accordingly, we propose that psychotherapy should be integrated into the treatment of PD patients to enhance their quality of life and discourage them from discontinuing ongoing medical therapies.

## Figures and Tables

**Table 1:** Demographic characteristics of 564 patients with Peyronie’s disease.

Demographic Characteristics	N. Patients (out of 564) (%)
Race/Ethnicity	
Caucasian	561 (99.1)
Indian	1 (0.17)
Pakistani	1 (0.17)
Kurdish	1 (0.17)
Arab	1 (0.17)
Armenian	1 (0.17)
Age range (years)	N. patients (out of 564) (%)
21–30	51 (9.0)
31–40	86 (15.2)
41–50	130 (23.0)
51–60	183 (32.4)
61–70	104 (18.4)
71–74	10 (1.7)
Type of school education	N. patients (out of 564) (%)
Elementary school	16 (2.8)
Secondary school	413 (73.2)
University degree	135 (23.9)
Type of employment	N. patients (out of 564) (%)
Artist/dancer	1 (0.17)
Employee	318 (56.34)
Teacher	45 (7.9)
Physician	10 (1.7)
Psychologist	5 (0.88)
Business manager	59 (10.4)
Journalist	4 (0.7)
Construction worker	3 (0.5)
Agricultural worker	3 (0.5)
Student	20 (3.5)
Pensioner	53 (9.3)
Unemployed	43 (7.6)
Marital status	N. patients (out of 564) (%)
Married	265 (46.9)
Unmarried	299 (53.0)

**Table 2:** Clinical features of 564 patients with Peyronie’s disease.

Clinical Conditions Associated			N. Patients (out of 564) (%)		
Diabetes mellitus			32 (5.6)		
Hypertension			102 (18.0)		
Benign prostatic hyperplasia			120 (21.2)		
Prostatitis			201 (35.6)		
Hyperlipemia			59 (10.4)		
Hyperhomocysteinemia			10 (1.7)		
Obesity			31 (5.4)		
Hypothyroidism			27 (4.7)		
Hyperthyroidism			1 (0.17)		
Dupuytren disease			34 (6.0)		
Ledderhose disease			15 (2.6)		
Autoimmune diseases			48 (8.5)		
Previous acute myocardial infarction			14 (2.4)		
Arteriopathy (carotid, femoral, aorta)			14 (2.4)		
Previous endourological maneuvers *			26 (4.6)		
Previous radical retropubic prostatectomy (RRP)			5 (0.8)		
History of recent penile trauma			164 (29.0)		
Excessive consumption of alcohol **			40 (7.0)		
Cigarette smoking (≥10 daily)			86 (15.2)		
Primary symptoms of Peyronie’s disease	N. patients (of 564) (%)	Value used	Value range	Mean value	Standard deviation (SD) ±
Penile curvature Penile deformity (excluding curvature)	511 (90.6) 508 (90.0)	Degrees -	5–100 -	35.4 -	±17.5

Penile pain	301 (53.3)	VAS score	0–10	4.3	±1.8
Erectile dysfunction (ED)	225 (39.8)	IIEF score	0–30	21.5	±2.7
Anxiety	499 (88.4)	GAD-7 score	10–21	15.9	±3.5
Depression	325 (57.62)	PHQ-9 score	0–27	14.1	±3.2
Psychosexual impact (PDQ bother)	527 (93.4)	PDQ score	0–16	8.8	±2.7

NOTE: * Previous endourological maneuvers = transurethral resection of prostate (TURP) or bladder tumors (TURBs), urethral catheterization, ureteroscopy, or cystoscopy; ** wine consumption > 500 mL per day and/or habitual consumption of hard liquor; VAS = visual analog scale, questionnaire for measuring pain [56]; IIEF = International Index of Erectile Function, questionnaire for erectile function measurement, where the score ranges from 0 to 30, with the following interpretations: severe ED (0–10), moderate ED (11–16), mild to moderate ED (17–21), mild ED (22–25), and ED (26–30) [57]; GAD-7 = Generalized Anxiety Disorder-7 questionnaire, for anxiety measurement, where “significant anxiety” is present when GAD-7 score > 9 [58]; PHQ-9 = Patient Health Questionnaire-9, for depressive measurement, where significant depression is present when PHQ-9 score > 9 [59]; PDQ bother = Peyronie’s Disease Questionnaire/symptom bother to evaluate the psychosexual impact, where the score ranges from 0 to 30 [60,61]. The onset of the disease had occurred between 6 and 26 months previously. Mean time elapsed since the onset of the disease = 11.5 months, standard deviation (SD) ± 2.1 months.

**Table 3:** Findings related to the time of ED presentation in the 225 PD patients.

Erectile Dysfunction (ED)	N. Patients out of 225 (%)	IIEF Score Mean Value	Standard Deviation (SD) ±
Total number of cases with ED	225 (100)	21.5	±2.7
Cases with ED onset during PD	98 (43.5)	22.3	±2.6
Cases with ED present before the onset of PD	127 (56.4)	20.9	±2.7

**Table 4:** Findings related to the ultrasound appearance, palpation characteristics, and volume and location of the penile plaque in 564 patients with Peyronie’s disease.

Palpatory Features of the Plaque	N. Cases (out of 564) (%)
Non-palpable	62 (10.9)
Palpable	502 (89.0)
Palpable as soft	25 (4.4)
Palpable as fibrous	280 (49.6)
Palpable as fibrocalcific	197 (34.9)
Ultrasound imaging of the plaque	N. cases (out of 564) (%)
Hypo-isoechoic	27 (4.7)
Isoechoic	23 (4.0)
Hyperechogenicity	306 (54.2)
Hypoisoecogenicity associated with hyperechogenicity (mixed plaque)	5 (0.8)
Isohyperechogenicity (mixed plaque)	2 (0.35)
Hypoisoecogenicity associated with calcification (mixed plaque)	13 (2.3)
Isohyperechogenicity associated with calcification (mixed plaque)	8 (1.4)
Hyperechogenicity associated with calcification (mixed plaque)	180 (31.9)
Penile plaque with internal calcification	201 (35.6)
Plaque with calcification (classification) *	N. cases (out of 201) (%)
Grade 1 = punctiform or ≤3 mm	23 (11.4)
Grade 2 = >3 mm and <15 mm	130 (64.6)
Grade 3 = ≥15 mm or ≥2 plaque >10 mm	48 (23.8)
Plaque volume (mm^3^)	N. cases (out of 564) (%)
Up to 100	12 (2.1)
From 101 to 300	54 (9.5)
From 301 to 500	114 (20.2)
From 501 to 1000	209 (37.0)
From 1001 to 2000	142 (25.1)
From 2001 to 3000	28 (4.9)
>3000	5 (0.8)
Penile location of the plaque	N. cases (out of 564) (%)
Basal third	70 (12.4)
Middle third	124 (21.9)
Distal third	171 (30.3)
Middle third and distal third	81 (14.3)
Basal + middle third	10 (1.7)
Basal third + distal third	65 (11.5)
Middle third + distal third	16 (2.8)
Basal and middle third	25 (4.4)
From basal third to distal third	2 (0.3)

Note: * Classification of calcifications according to Levine et al. [62]: Grade 1 = punctiform or ≤3 mm; Grade 2 = >3 mm and <15 mm; Grade 3 = ≥15 mm or ≥2 plaque >10 mm.

**Table 5:** Penile deformity: prevalence, typology, and characteristics in 564 patients with Peyronie’s disease.

Penile Curvature		N. Cases out of 564 (%)	Degrees of Penile Curvature from x to x Degrees		Mean of Curvature Degrees	Standard Deviation ±
		511 (90.6)	5–100		35.47	±17.5
Categorization based on their severity	N. cases Out of 511 (%)	5–12	15–28		30–58	60–100
		46 (9.0)	103 (20.1)		314 (61.4)	48 (9.32)
Type of penile curvature		N. cases out of 511 (%)				
Dorsal		160 (31.3)	10–90		38.4	±16.7
Lateral to the left		143 (27.8)	5–100		32.7	±19.3
Lateral to the right		38 (7.4)	5–75		26.3	±15.7
Ventral		9 (1.7)	5–35		20.8	±10.3
Multiplanar curvature, dorsal, and left lateral		122 (23.8)	5–90		38.9	±15.9
Multiplanar curvature, dorsal, and right lateral		18 (3.5)	10–75		42.7	±16.6
Multiplanar curvature, ventral, and left lateral		11 (2.1)	30–50		29.3	±11.5
Multiplanar curvature, ventral, and right lateral		3 (0.58)	30–35		31.6	±2.8
Multiplanar curvature, to the right and left		2 (0.39)	25–30		27.5	±3.5
Multiplanar curvature, left lateral, right lateral, and ventral		3 (0.58)	5–45		20.0	±21.7
Multiplanar curvature, dorsal, left lateral, and right lateral		2 (0.39)	20–25		22.5	±3.5
Total number of Penile deformities (excluding curvatures)		N. cases 508 90.0% of 564				
Type of penile deformity		N. cases (out of 564) (%)	Associated with penile curvature N. cases	Not associated with penile curvature N. cases	Associated with other penile deformity N. cases	Isolated deformity N. cases
Penile shortening *		498 (88.2)	453	45	119	379
Flail penis **		2 (0.35)	2	0	2	0
Penile hourglass		69 (12.2)	58	11	65	4
Penile divot		44 (7.8)	26	18	39	5
Penile torsion		13 (2.3)	9	4	12	1
Solitary penile deformities (not curvature)		389 (68.9)				
Associated penile deformities in the same patient ***		119 (21.0)				
Total number of patients with penile deformation (not curvature)		508 (90.07)				
Total number of patients without penile deformation (including curvature)		53 (9.39)				

NOTE: * Penile shortening ranged from 1 to 4.5 cm. In all cases, the plaque volume was greater than 300 mm^3^. ** “Flail” penis is a term used to describe a situation in which the penis, in an erect state, is soft, flaccid, and mobile in the distal area, compared to the more proximal part with normally rigid tissue. *** More than one penile deformity occurring in the same patient, where the associated deformities were always of two types.

**Table 6:** Prevalence of anxious–depressive state in 564 patients with Peyronie’s disease.

	GAD-7 Score Range	No. Total Cases (%)
No anxiety	0	2 (3.5)
Minimal or mild anxiety	1–9	63 (11.1)
Moderate anxiety	10–14	284 (49.6)
Severe anxiety	15–21	219 (38.8)
Total number of cases with anxiety status	3–21	562 (99.6)
Significant anxiety	10–21	499 (88.4)
	PHQ-9 score range	
No depression	0	2 (3.5)
Minimal or mild depression	1–9	237 (42.0)
Moderate depression	10–14	205 (36.3)
Moderately severe depression	15–19	95 (16.8)
Severe depression	20–27	25 (4.4)
Total number of cases with depression status	1–27	562 (99.6)
Significant depression	10–27	325 (57.6)

NOTE: GAD-7 = Generalized Anxiety Disorder-7 questionnaire, for anxiety disorder; PHQ-9 = Patient Health Questionnaire-9, for depressive disorder. Significant anxiety is present when GAD-7 score > 9 [58]. Significant depression is present when PHQ-9 score >9 [59].

**Table 7:** List of possible statistical correlations between the different variables.

Statistical Correlation Analysis				Pearson Correlation Coefficient (r)	*p*-Value	Correlation Present? YES or NO
Between	VAS score	and	Age	−0.207	0.0003	YES
Between	Degree of penile curvature	and	Age	0.205	0.000002	YES
Between	Plaque volume	and	Time elapsed since the disease onset	0.832	<0.0001	YES
Between	Degree of penile curvature	and	Time elapsed since the disease onset	0.175	0.00006	YES
Between	IIEF score	and	Time elapsed since the disease onset	0.0249	0.554	NO
Between	VAS score	and	Time elapsed since the disease onset	0.0545	0.195	NO
Between	Degree of penile curvature	and	Plaque volume	0.171	0.00004	YES
Between	IIEF score	and	Plaque volume	0.070	0.09	NO
Between	IIEF score	and	Age	−0.238	0.000009	YES
Between	GAD-7 score	and	PHQ-9 score	0.667	<0.0001	YES
Between	GAD-7 score	and	PDQ bother score	0.907	<0.0001	YES
Between	PHQ-9 score	and	PDQ bother score	0.612	<0.0001	YES
Between	GAD-7 score	and	Severity of penile curvature	0.132	0.002	YES
Between	PHQ-9 score	and	Severity of penile curvature	0.192	0.0001	YES
Between	PDQ bother score	and	Severity of penile curvature	0.178	0.00005	YES
Between	VAS score	and	GAD-7 score	0.154	0.007	YES
Between	VAS score	and	PHQ-9 score	0.260	0.000004	YES
Between	VAS score	and	PDQ bother score	0.137	0.017	YES
Between	IIEF score	and	GAD-7 score	−0.303	<0.001	YES
Between	IIEF score	and	PHQ-9 score	−0.060	0.151	NO
Between	IIEF score	and	PDQ bother score	−0.185	0.000	YES
Between	IIEF score	and	Severity of penile curvature	−0.063	0.359	NO
Between	IIEF score	and	VAS score	0.069	0.483	NO

## Data Availability

The original contributions presented in the study are included in the article, further inquiries can be directed to the corresponding author/s.
